# Receptive and Expressive Vocabulary Skills and Their Correlates in Mandarin-Speaking Infants with Unrepaired Cleft Lip and/or Palate

**DOI:** 10.3390/ijerph17093015

**Published:** 2020-04-26

**Authors:** Si-Wei Ma, Li Lu, Ting-Ting Zhang, Dan-Tong Zhao, Bin-Ting Yang, Yan-Yan Yang, Jian-Min Gao

**Affiliations:** 1 Clinical Research Center of Shaanxi Province for Dental and Maxillofacial Diseases, College of Stomatology, Xi’an Jiaotong University, Xi’an 710004, China; masiwei@xjtu.edu.cn (S.-W.M.); yangbinting1221@sina.com (B.-T.Y.); yangyanyan9@sina.com (Y.-Y.Y.); 2 School of Public Health, Xi’an Jiaotong University Health Science Center, Xi’an 710061, China; 3 Research Center of Stomatology, College of Stomatology, Xi’an Jiaotong University, Xi’an 710004, China; 4 Team IETO, Bordeaux Population Health Research Center, UMR U1219, INSERM, Université de Bordeaux, 33076 Bordeaux, France; liser@outlook.com; 5 Department of English, Faculty of Arts and Humanities, University of Macau, Taipa, Macao 999078, China; tingtingzhang90@hotmail.com; 6 School of Public Policy and Administration, Xi’an Jiaotong University, Xi’an 710049, China; zdt0102@stu.xjtu.edu.cn

**Keywords:** China, vocabulary skills, receptive vocabulary, expressive vocabulary, Mandarin, cleft lip and/or palate, infants

## Abstract

Background: Vocabulary skills in infants with cleft lip and/or palate (CL/P) are related to various factors. They remain underexplored among Mandarin-speaking infants with CL/P. This study identified receptive and expressive vocabulary skills among Mandarin-speaking infants with unrepaired CL/P prior to cleft palate surgery and their associated factors. Methods: This is a cross-sectional study involving patients at the Cleft Lip and Palate Center of the Stomatological Hospital of Xi’an Jiaotong University between July 2017 and December 2018. The Putonghua Communicative Development Inventories-Short Form (PCDI-SF) was used to assess early vocabulary skills. Results: A total of 134 children aged 9–16 months prior to cleft palate surgery were included in the study. The prevalences of delays in receptive and expressive vocabulary skills were 72.39% (95% CI: 64.00–79.76%) and 85.07% (95% CI: 77.89–90.64%), respectively. Multiple logistic regression identified that children aged 11–13 months (OR = 6.46, 95% CI: 1.76–23.76) and 14–16 months (OR = 24.32, 95% CI: 3.86–153.05), and those with hard/soft cleft palate and soft cleft palate (HSCP/SCP) (OR = 5.63, 95% CI: 1.02–31.01) were more likely to be delayed in receptive vocabulary skills. Conclusions: Delays in vocabulary skills were common among Mandarin-speaking CL/P infants, and age was positively associated with impaired and lagging vocabulary skills. The findings suggest the necessity and importance of early and effective identification of CL/P, and early intervention programs and effective treatment are recommended for Chinese CL/P infants.

## 1. Introduction

Cleft lip and/or palate (CL/P) is one of the most common congenital anomalies. The global live births incidence is 1.2/1000 [1]. The incidence in China is 1.48–3.27/1000 live births [2,3], which is greater than that in other regions, such as North America (1.56/1000), Europe (1.55/1000), Oceania (1.33/1000), South America (0.99/1000), and Africa (0.57/1000) [4,5,6]. Cleft palate speech is known as one of the most mysterious communication disorders. Because of the intricate interactions between surgical intervention, speech and language skills and the great variability in craniofacial morphology, even patients who are diagnosed with the same type of cleft lip and palate could exhibit different responses to the treatment [7]. As a result, the treatment of cleft palate speech is far from simple and clear, but calls for further investigation [8,9].

Phonetic learning (i.e., acquiring pronunciation information about words) and semantic learning (i.e., learning the meanings of language symbols, like sounds and text) are inseparable in the process of language acquisition [10,11]. Early word learning plays an important role in shaping the phonological system among infants aged 7–17 months, which is also a functional reorganization period [12,13]. Additionally, infants at this age group experienced a transition from a stage of sensitivity to phonetic details in syllables to a stage of reorganization of phonetic information, in which infants demonstrated difficulty with discriminating phonetically similar words [12]. In the process of mapping phonetic information to meaning—that is, to the corresponding referent in the context, such as an object, a person, or things that the infant sees, hears, or even asks for—infants also constantly develop their capacity for phoneme processing and, further, gradually establish their native language system, in particular the phonological system [10]. This is a critical period for early development of the phonological system, during which infants with cleft palate generally receive the most important intervention, i.e., the cleft palate operation. The impact of cleft palate on the early development of children’s sound system occurs far earlier than 12 months of age [14]. Research on speech sounds and syllabic features in babbling has demonstrated that, in the prelinguistic stage, compared to typically developing (TD) children, infants with CL/P are more likely to have a relatively late babbling stage, and their frequency of babbling is also not as high as the TD children [15,16,17]. In addition, children with CL/P normally have fewer oral plosives and apical consonants, but produce more nasals and glottal sounds [18,19,20].

More interestingly, unlike TD children, the transitions from nonverbal communication to verbal perception and production are delayed, and their accumulation of the consonants inventory is usually not sufficient [21]. Some studies have found the receptive language skills of infants with CL/P to be “typical” compared with non-cleft infants of the same age [22,23]. With the assessment of standardized tests, studies found that infants with CL/P also suffered from a delayed development of expressive language skills [23,24,25,26,27]. Various factors may contribute to the delay of early vocabulary skills in children with CL/P, including anatomical differences, phonological disorders, central nervous system dysfunction and sociocultural impacts [23,28,29,30].

The Mandarin syllable structure is relatively simple (C_0–1_VC_0–1_ + Tone); CL/P infants are more likely to resolve their articulation difficulties through the omission of initial consonants, which is similar to Cantonese-speaking CL/P children [31]. This may play a role in limiting recognition of their speech by caregivers. Mandarin-speaking CL/P children may face more challenges than their English-speaking peers.

Surgeons, speech pathologists, and members of cleft palate/craniofacial teams have been trying for decades to gain some kind of “sure grip” on the relationship between age at palatoplasty and children’s speech development [9]. In China, 9–12 months is considered the optimal age period for palatoplasty [32]. However, it may be very difficult for some patients to take the treatment at an appropriate time, due to many potential factors, such as the great gap in economic status and obtaining of medical information regarding cleft treatment between urban and rural patients. Research on speech and language pathology in mainland China is still rather primitive, and the field lacks language assessment tools with satisfactory validity and reliability [33]. More importantly, compared to children with CL/P who have had the palatal surgery, the language skills of children with CL/P at the pre-surgery stage have received less attention [23] and deserve more investigation. Besides, studies on the vocabulary skills of Mandarin-speaking infants with CL/P are even more sparse [26]. 

Therefore, in order to further provide a research basis for early intervention programs for Mandarin-speaking infants with CL/P, this study aimed at (1) identifying the receptive and expressive vocabulary skills among Mandarin-speaking infants with unrepaired CL/P; and (2) explore associations between their sociodemographic and clinical characteristics and vocabulary skills. We hypothesized that receptive and expressive vocabulary skills among infants with unrepaired CL/P could lag behind standardized norms, and age could be positively associated with impaired and delayed vocabulary skills. 

## 2. Materials and Methods 

### 2.1. Settings, Subjects and Data Collection

This retrospective study patients at the Cleft Lip and Palate Center of Stomatological Hospital of Xi’an Jiaotong University between July 2017 and December 2018. Cleft lip and palate patients were diagnosed according to ICD-10 (Code: Q35, Q37) and classified according to the Veau’s simplified classification of palatal clefts [34]. Infants with unrepaired cleft lip and/or palate before palatal surgery and aged 8–16 months were included. Those with a clear diagnosis of other comorbidities (such as syndrome clefts) or stunted growth were excluded. The study protocol was approved by the Ethical Committee of Xi’an Jiaotong University Health Science Center (protocol number: 2019-754).

### 2.2. Assessment

Basic sociodemographic and clinical characteristics, including sex, months of age (age in months was equal to age at operation; age in months was coded into clinically relevant age groups: 8–10/11–13/14–16 months [35,36]), type of clefts, province (Shaanxi/others), maternal and paternal education level, and main companion–infant interaction time (hours/day) were extracted from medical records. 

Receptive and expressive vocabulary skills were assessed using the Putonghua Communicative Development Inventories-Short Form (PCDI-SF) of vocabulary and gestures forms (Cronbach’s alpha: 0.84–0.99) [37]. The Putonghua MacArthur–Bates Communicative Development Inventories (PCDI) [38] were adapted from the MacArthur–Bates Communicative Development Inventories (MCDI) [39] and were validated in Chinese infants aged 8–30 months [40]. It is a parental reporting assessment, which consists of two parts: one for children from 8 to 16 months (vocabulary and gestures) and another for 16–30 months (vocabulary and sentences). There are three parts in the PCDI-SF for 8 to 16 months, including “response to language in the early years”, “actions and gestures”, and “vocabulary”. Parents were required to complete this questionnaire upon admission. In this study, we used 106 items regarding “vocabulary”. Whether the “receptive vocabulary skills” and “expressive vocabulary skills” lag behind (delays in vocabulary skills) was screened and evaluated by comparing with the standardized norms of the child’s vocabulary development level. If the receptive and expressive vocabulary size were less than the age-equivalent size of the 50th percentile of the standardized norms, the participants would be rated as “delay” [37]. 

### 2.3. Data Analysis 

Statistical analyses were performed using STATA version 13.0 (Stata Corporation, College Station, TX, USA). The sociodemographic and clinical characteristics of those whose receptive vocabulary skills were and were not delayed, and those whose expressive vocabulary skills were and were not delayed, were compared by using a chi-square test. 

Multiple logistic regression analyses were conducted by setting delays in receptive vocabulary skills and delays in expressive vocabulary skills as the dependent variables, separately; sex, age in months, type of cleft, province of residence, maternal education level, paternal education level and interaction time were the independent variables. The significance level was set at 0.05 (two-tailed).

## 3. Results

### 3.1. Basic Characteristics of the Participants

A total of 134 children before cleft palate surgery were included in the study. The children were aged between 9 and 16 months (12.43 ± 1.77 months), and 48.51% (*n* = 65) of them were girls. Table 1 displays the sociodemographic and clinical characteristics of the whole sample and separately by delays in receptive and expressive vocabulary skills. The prevalences of delays in receptive vocabulary skills and expressive vocabulary skills were 72.39% (95% CI: 64.00–79.76%) and 85.07% (95% CI: 77.89–90.64%), respectively. A total of 64.18% (*n* = 84, 95% CI: 55.44–72.27%) of CL/P infants had delays in both receptive and expressive vocabulary. 

The univariate analyses showed that age significantly differed with lagging behind in expressive vocabulary skills (X^2^ = 12.97, *p* = 0.002). A significant difference was found in main companion–infant interaction hours (X^2^ = 4.71, *p* = 0.03) with lagging behind in receptive vocabulary skills. 

### 3.2. Associations between Expressive and Receptive Vocabulary Skills and Sociodemographic and Clinical Factors

Multiple logistic regression showed that infants aged 11–13 months (OR = 6.46, 95% CI: 1.76–23.76), 14–16 months (OR = 24.32, 95% CI: 3.86–153.05) and with HSCP/SCP (OR = 5.63, 95% CI: 1.02–31.01) were more likely to lag behind the norms in expressive vocabulary skills (Table 2). Longer main companion–infant interaction hours (>3 h/day) showed weak evidence of a negative correlation (OR = 0.45, 95% CI: 0.20–1.002) with delays in receptive vocabulary skills. 

## 4. Discussion

This study identified vocabulary skills and explored their correlates based on a sample of Mandarin-speaking infants with unrepaired CL/P. The results indicated that (1) 72.39% (95% CI: 64.00–79.76%) and 85.07% (95% CI: 77.89–90.64%) of CL/P infants aged 8–16 months lagged behind the norms in receptive and expressive vocabulary skills; (2) compared to those aged 8–10 months and with bilateral cleft lip/palate, infants aged between 11–16 months and with hard and/or soft cleft palate were more likely to lag behind the norms in expressive vocabulary skills. 

Early speech sound development might be significantly impaired before palatal surgery among infants with CL/P [14], as we found high proportions of CL/P infants lagging behind the norms in early receptive and expressive language skills. A study that used the same screening tool, i.e., the Putonghua MacArthur–Bates Communicative Development Inventories (PCDI), showed that Chinese infants with CL/P began to fall in the lexical development behind their non-cleft peers from 14 to 15 months [26]. It has been reported that the later CL/P infants take the palatal surgery, the greater the risk of sluggish vocabulary skills [9]. The Lancet series on early childhood development has already illustrated the necessity of preventative interventions to ensure children reach their developmental potential [41,42]. However, there is a shortage of at least 100,000 Speech-Language Pathologists (SLPs) in China [43], and the number of multidisciplinary cleft lip and palate therapy centers are very limited [44]. Therefore, training programs and allocation of Chinese SLPs, early identification and intervention programs, and treatment for vocabulary skills delay among Chinese infants with CL/P should be of concern. 

We found that the risks of lagging in expressive vocabulary skills among infants aged 14–16 months and 11–13 months were nearly 24 times and 6.5 times that of infants aged 8–10 months. A delay in early expressive vocabulary skills was also reported in English-speaking babies with CL/P [23]. One study that used CDI (parent report) found that lower scores in vocabulary production are maintained in cleft palate children from 16 to 30 months [22]. Another study found that infants with CP exhibited a 3-month delay in word acquisition compared with the non-cleft at 15, 18, and 21 months [45]. Compared with English, the syllable structure in Mandarin is simpler, as no consonant clusters are permitted and only the nasals /n/ and /ŋ/ are allowed as final consonants. Syllable-initial and syllable-final consonant inventories are asymmetrical [46], which could partly explain the discrepancies with our results. However, whether the phonological features of Mandarin could lead to a lagging risk in early word-learning development among Mandarin-speaking infants and toddlers with CL/P requires further cross-linguistics studies. 

We found that infants with HSCP/SCP (OR = 5.63, 95% CI: 1.02–31.01) were positively associated with lagging behind the norms in early expressive vocabulary skills. The Veau’s simplified classification of palatal clefts was used in this study, which includes bilateral cleft lip/palate (BCLP), unilateral cleft lip/palate (UCLP), hard/soft cleft palate (HSCP) and soft cleft palate (SCP) [47]. It should be noted that few researchers have examined the impacts of cleft type on early language skills [48], especially in China. One Chinese team studied early Mandarin vocabulary development involving 19 CLP infants and 21 CPO (i.e., HSCP and SCP in Veau’s classification) infants compared with the non-cleft peers, but without exploring the associations between type of cleft palate and early language skills [26]. Studies from other countries, such as the USA, showed that at the age of 30 months, the CPO group performed worse than the CLP group on receptive and expressive language skills [19], which was consistent with our results even though focusing on different age populations. However, another study in the USA using the Bayley Scales of Infant Development (BSID) items showed that infants with CLP (*n* = 29) and CPO (*n* = 28) did not differ on the score of nonverbal and expressive language skills [49]. Disparities in methodology and limited sample sizes could partly explain the differences among various cleft type sub-groups in existing studies [17,48]; further studies with larger sample sizes are warranted. 

A longer main companion–infant interaction time (>3 h/day) showed weak evidence of a negative correlation (OR = 0.45, 95% CI: 0.20–1.002) with lagging in early receptive language skills in the early word-learning period. The amount of parent–child interaction has been related to early language skills from a very young age (8–10 months) [50]. The TD children happen to experience a leap from prelinguistic to emerging language, but the processes by which language skills are supported by caregivers in the natural environment may be interfered with by the early pronunciation characteristics of infants with CL/P [51]. The language skills of children with CL/P become more complicated along with increasing age and operation intervention [28]; further studies with regards to parent–child interaction should be conducted.

Sex, main companion–infant interaction hours, parents’ education and geographical location were not identified as correlates of early language skills in this study, which was demonstrated in some existing studies [52,53,54]. A previous study of Mandarin-speaking TD children has also revealed that environmental factors play a significant role in vocabulary development after 2 years of age [55].

Our study explored early receptive and expressive language skills of Mandarin-speaking infants with CL/P aged 8–16 months using a validated assessment tool. It suggested that the delay of early language skills in Chinese children with CL/P might emerge earlier, and the lagging in vocabulary skills might be unfavorable for Mandarin-speaking children. Surgical interventions should be considered early and promptly to prevent great damage in word learning among Mandarin-speaking infants with CL/P. 

However, there are some limitations. Firstly, the data was from a single cleft lip and palate treatment center rather than multi-centers, which could limit the generalization. Second, a parental report instrument, i.e., the abbreviated version of CDI, was used to evaluate early language skills; the CDI and its short form have been widely used in clinical research [56,57,58,59]. In addition, other characteristics, such as cognitive ability of included infants, which could be related factors, were not assessed and analyzed. Lastly, we only evaluated delay, without exploring degrees of delay in early receptive and expressive language skills, which should be further studied. 

## 5. Conclusions

In conclusion, the present study identified that lagging behind in receptive and expressive vocabulary skills was common among Mandarin-speaking infants with unrepaired cleft lip and/or palate. There were associations between age and type of cleft and vocabulary skills, which could help to better target early screening and intervention programs, CL/P infants with late operation need more efficient intervention. Further studies are also necessary, since the operation time should be determined by considering a number of other factors.

## Figures and Tables

**Table 1:** Sociodemographic and clinical characteristics of the included infants.

Variables	Categories	Total (*n* = 134)		Delays in Receptive Vocabulary Skills (*n* = 97)			Delays in Expressive Vocabulary Skills (*n* = 114)		
		*n*	%	*n*	%	X^2^ (*p*)	*n*	%	X^2^ (*p*)
Sex	Male	69	51.5	50	51.5	0.0004 (0.984)	62	54.3	2.58 (0.108)
	Female	65	48.5	47	48.5		52	45.6	
Age (months)	8–10	18	13.4	14	14.4	3.66 (0.16)	10	8.8	12.97 (**0.002**)
	11–13	77	57.5	51	52.6		67	58.8	
	14–16	39	29.1	32	33.0		s	32.5	
Type of clefts	BCLP	13	9.7	9	9.3	0.41 (0.814)	10	8.8	0.88 (0.642)
	HSCP/SCP *	87	64.9	62	63.9		74	64.9	
	UCLP	34	25.4	26	26.8		30	26.3	
Province of residence	Shaanxi	93	69.4	65	67.0	0.97 (0.324)	80	70.2	0.21 (0.647)
	Others	41	30.6	32	33.0		34	29.8	
Maternal education	High school and below	75	56.0	54	55.7	0.01 (0.91)	65	57.0	0.34 (0.561)
	College and above	59	44.0	43	44.3		49	43.0	
Paternal education	High school and below	77	57.5	55	56.7	0.08 (0.772)	67	58.8	0.53 (0.466)
	College and above	57	42.5	42	43.3		47	41.2	
Interaction time (hours)	≤3 h	85	63.4	67	69.1	4.71 (**0.03**)	72	63.2	0.03 (0.874)
	>3 h	49	36.6	30	30.9		42	36.8	

*: There were two infants with SCP in the sample, so HSCP and SCP were merged. BCLP = bilateral cleft lip/palate; UCLP = unilateral cleft lip/palate; HSCP = hard/soft cleft palate; SCP = soft cleft palate. Bold values: *p* < 0.05.

**Table 2:** Multiple logistic regressions on the associations between sociodemographic and clinical characteristics and vocabulary skills (*n* = 134).

Variables	Categories	Delays in Receptive Vocabulary Skills			Delays in Expressive Vocabulary Skills		
		OR	95% CI		OR	95% CI	
			Lower	Upper		Lower	Upper
Sex	Male	1	-	-	1	-	-
	Female	1.05	0.43	2.51	0.36	0.11	1.22
Age (months)	8–10	1	-	-	1	-	-
	11–13	0.77	0.22	2.75	6.46	**1.76**	**23.73**
	14–16	1.67	0.39	7.15	24.32	**3.86**	**153.05**
Type of clefts	BCLP	1	-	-	1	-	-
	HSCP/SCP	1.33	0.33	5.30	5.63	**1.02**	**31.01**
	UCLP	1.56	0.33	7.47	5.45	0.65	45.43
Province of residence	Shaanxi	1	-	-	1	-	-
	Others	1.50	0.43	2.52	0.84	0.22	3.18
Maternal education	High school and below	1	-	-	1	-	-
	College and above	0.99	0.33	2.99	0.66	0.16	2.70
Paternal education	High school and below	1	-	-	1	-	-
	College and above	1.04	0.65	1.67	1.03	0.57	1.86
Interaction time	≤3 h	1	-	-	1	-	-
	>3 h	0.45	0.20	1.002	1.01	0.32	3.16

BCLP = bilateral cleft lip/palate; UCLP = unilateral cleft lip/palate; HSCP = hard/soft cleft palate; SCP = soft cleft palate. Bold values: *p* < 0.05.

## References

[1] Rahimov F., Jugessur A., Murray J.C. (2012). Genetics of Nonsyndromic Orofacial Clefts. Cleft Palate Craniofacial J..

[2] Chang W.J., See L.C., Lo L.J. (2016). Time Trend of Incidence Rates of Cleft Lip/Palate in Taiwan from 1994 to 2013. Biomed. J..

[3] Li Z., Ren A., Liu J., Zhang L., Ye R., Li S., Li Z. (2008). High Prevalence of Orofacial Clefts in Shanxi Province in Northern China, 2003–2004. Am. J. Med. Genet. Part A.

[4] Mossey P.A., Little J., Munger R.G., Dixon M.J., Shaw W.C. (2009). Cleft Lip and Palate. Lancet.

[5] Dixon M.J., Marazita M.L., Beaty T.H., Murray J.C. (2011). Cleft Lip and Palate: Understanding Genetic and Environmental Influences. Nat. Rev. Genet..

[6] Panamonta V., Pradubwong S., Panamonta M., Chowchuen B. (2015). Global Birth Prevalence of Orofacial Clefts: A Systematic Review. J. Med. Assoc. Thail. Chotmaihet Thangphaet.

[7] Molsted K. (1999). Treatment Outcome in Cleft Lip and Palate: Issues and Perspectives. Crit. Rev. Oral Biol. Med..

[8] Shprintzen R.J. (2013). The Velopharyngeal Mechanism. Cleft Lip Palate Diagn. Manag..

[9] Peterson-Falzone S.J. (2013). Optimal Age for Palatoplasty to Facilitate Normal Speech Development: What Is the Evidence?. Cleft Lip Palate Diagn. Manag..

[10] Cao M., Li A., Fang Q., Kröger B.J. Phonetic-Phonological Feature Emerges by Associating Phonetic with Semantic Information—A GSOM-Based Modeling Study. Proceedings of the Sixteenth Annual Conference of the International Speech Communication Association, INTERSPEECH.

[11] Kröger B.J., Crawford E., Bekolay T., Eliasmith C. (2016). Modeling Interactions between Speech Production and Perception: Speech Error Detection at Semantic and Phonological Levels and the Inner Speech Loop. Front. Comput. Neurosci..

[12] Stager C.L., Werker J.F. (1997). Infants Listen for More Phonetic Detail in Speech Perception than in Word-Learning Tasks. Nature.

[13] Werker J.F., Fennell C.T., Corcoran K.M., Stager C.L. (2002). Infants’ Ability to Learn Phonetically Similar Words: Effects of Age and Vocabulary Size. Infancy.

[14] Peterson-Falzone S.J., Trost-Cardamone J.E., Karnell M.P., Hardin-Jones M.A. (2017). The Clinician’s Guide to Treating Cleft Palate Speech.

[15] Ha S. (2018). Profiles of Vocal Development in Korean Children with and without Cleft Palate. Clin. Linguist. Phon..

[16] Scherer N.J., Boyce S., Martin G. (2013). Pre-Linguistic Children with Cleft Palate: Growth of Gesture, Vocalization, and Word Use. Int. J. Speech Lang. Pathol..

[17] Chapman K.L., Hardin-Jones M., Schulte J., Halter K.A. (2001). Vocal Development of 9-Month-Old Babies with Cleft Palate. J. Speech Lang. Hear. Res..

[18] Lohmander A., Olsson M., Flynn T. (2011). Early Consonant Production in Swedish Infants with and without Unilateral Cleft Lip and Palate and Two-Stage Palatal Repair. Cleft Palate Craniofacial J..

[19] Scherer N.J., Williams A.L., Proctor-Williams K. (2008). Early and Later Vocalization Skills in Children with and without Cleft Palate. Int. J. Pediatric Otorhinolaryngol..

[20] Jones C.E., Chapman K.L., Hardin-Jones M.A. (2003). Speech Development of Children with Cleft Palate before and after Palatal Surgery. Cleft Palate Craniofacial J..

[21] Hardin-Jones M., Chapman K.L. (2014). Early Lexical Characteristics of Toddlers with Cleft Lip and Palate. Cleft Palate Craniofacial J..

[22] Scherer N.J., D’Antonio L.L. (1995). Parent Questionnaire for Screening Early Language Development in Children with Cleft Palate. Cleft Palate Craniofacial J..

[23] Hardin-Jones M., Chapman K.L. (2011). Cognitive and Language Issues Associated with Cleft Lip and Palate. Semin. Speech Lang..

[24] Snyder L.E., Scherer N. (2004). The Development of Symbolic Play and Language in Toddlers with Cleft Palate. Am. J. Speech Lang. Pathol..

[25] Young S.E., Purcell A.A., Ballard K.J. (2010). Expressive Language Skills in Chinese Singaporean Preschoolers with Nonsyndromic Cleft Lip and/or Palate. Int. J. Pediatric Otorhinolaryngol..

[26] Lu Z., Ma L., Luo Y., Fletcher P. (2010). The Effects of Unrepaired Cleft Palate on Early Language Development in Chinese Infants. Cleft Palate Craniofacial J..

[27] Cavalheiro M.G., Lamônica D.A.C., de Vasconsellos Hage S.R., Maximino L.P. (2019). Child Development Skills and Language in Toddlers with Cleft Lip and Palate. Int. J. Pediatric Otorhinolaryngol..

[28] Kaiser A.P., Scherer N.J., Frey J.R., Roberts M.Y. (2017). The Effects of Enhanced Milieu Teaching With Phonological Emphasis on the Speech and Language Skills of Young Children With Cleft Palate: A Pilot Study. Am. J. Speech-Lang. Pathol..

[29] Shriver A.S., Canady J., Richman L., Andreasen N.C., Nopoulos P. (2006). Structure and Function of the Superior Temporal Plane in Adult Males with Cleft Lip and Palate: Pathologic Enlargement with No Relationship to Childhood Hearing Deficits. J. Child Psychol. Psychiatry Allied Discip..

[30] Morris H., Ozanne A. (2003). Phonetic, Phonological, and Language Skills of Children with a Cleft Palate. Cleft Palate Craniofacial J..

[31] Whitehill T., Stokes S., Hardcastle B., Gibbon F. (1995). Electropalatographic and Perceptual Analysis of the Speech of Cantonese Children with Cleft Palate. Eur. J. Disord. Commun..

[32] Shi B., Fu Y.C., Yin N.B. (2017). Application of Team Approach and Key Techniques of Cleft Lip and Palate. West China J. Stomatol..

[33] Liu X.L., de Villiers J., Ning C., Rolfhus E., Hutchings T., Lee W., Jiang F., Zhang Y.W. (2017). Research to Establish the Validity, Reliability, and Clinical Utility of a Comprehensive Language Assessment of Mandarin. J. Speech Lang. Hear. Res..

[34] Veau V. (1931). Division Palatine: Anatomie, Chirurgie, Phonétique.

[35] Mantel N., Haenszel W. (1959). Statistical Aspects of the Analysis of Data from Retrospective Studies of Disease. J. Natl. Cancer Inst..

[36] Marrinan E.M., Labrie R.A., Mulliken J.B. (1998). Velopharyngeal Function in Nonsyndromic Cleft Palate: Relevance of Surgical Technique, Age at Repair, and Cleft Type. Cleft Palate Craniofacial J..

[37] Tardif T., Fletcher P., Zhang Z.X., Liang W.L. (2008). The Chinese Communicative Development Inventory (Putonghua and Cantonese Versions): Manual, Forms, and Norms.

[38] Tardif T., Fletcher P., Liang W., Kaciroti N. (2009). Early Vocabulary Development in Mandarin (Putonghua) and Cantonese. J. Child Lang..

[39] Fenson L., Marchman V., Thal D., Dale P., Reznick J.S. (2007). MacArthur-Bates Communicative Development Inventories: User’s Guide and Manual.

[40] Tardif T. (2008). Chinese Communicative Development Inventories: User’s Guide and Manual.

[41] Black M.M., Walker S.P., Fernald L.C.H., Andersen C.T., DiGirolamo A.M., Lu C., McCoy D.C., Fink G., Shawar Y.R., Shiffman J. (2017). Early Childhood Development Coming of Age: Science through the Life Course. Lancet.

[42] Lo S., Das P., Horton R. (2017). A Good Start in Life Will Ensure a Sustainable Future for All. Lancet.

[43] Shan C. (2016). Seize the Opportunity to Realize a New Great Development in Speech-Language Pathology in China. Chin. J. Rehabil..

[44] Fu Y. (2017). Concept of Interdisciplinary Team Care for Cleft Lip and Palate. Interdisciplinary Team Care for Cleft Lip and Palate.

[45] Neiman G.S., Savage H.E. (1997). Development of Infants and Toddlers with Clefts from Birth to Three Years of Age. Cleft Palate Craniofacial J..

[46] Duanmu S. (2000). The Phonology of Standard Chinese.

[47] Allori A.C., Mulliken J.B., Meara J.G., Shusterman S., Marcus J.R. (2017). Classification of Cleft Lip/Palate: Then and Now. Cleft Palate Craniofacial J..

[48] Hardin-Jones M., Chapman K.L., Schulte J. (2003). The Impact of Cleft Type on Early Vocal Development in Babies with Cleft Palate. Cleft Palate Craniofacial J..

[49] Speltz M.L., Endriga M.C., Hill S., Maris C.L., Jones K., Omnell M.L. (2000). Cognitive and Psychomotor Development of Infants with Orofacial Clefts. J. Pediatric Psychol..

[50] Roberts M.Y., Kaiser A.P. (2011). The Effectiveness of Parent-Implemented Language Interventions: A Meta-Analysis. Am. J. Speech-Lang. Pathol..

[51] Frey J.R., Kaiser A.P., Scherer N.J. (2018). The Influences of Child Intelligibility and Rate on Caregiver Responses to Toddlers with and Without Cleft Palate. Cleft Palate Craniofacial J..

[52] Reilly S., Wake M., Bavin E.L., Prior M., Williams J., Bretherton L., Eadie P., Barrett Y., Ukoumunne O.C. (2007). Predicting Language at 2 Years of Age: A Prospective Community Study. Pediatrics.

[53] Law J., Rush R., Anandan C., Cox M., Wood R. (2012). Predicting Language Change between 3 and 5 Years and Its Implications for Early Identification. Pediatrics.

[54] Wallace I.F., Berkman N.D., Watson L.R., Coyne-Beasley T., Wood C.T., Cullen K., Lohr K.N. (2015). Screening for Speech and Language Delay in Children 5 Years Old and Younger: A Systematic Review. Pediatrics.

[55] Niu J., Chen Y.X., Zhu L.Q. (2015). Impacts of Biological and Family Factors on Lexical and Intellectual Development in Mandarin-Speaking Children. Chin. J. Contemp. Pediatrics.

[56] Kim S.W., Jeon H.R., Park E.J., Kim H.I., Jung D.W., Woo M.R. (2014). The Usefulness of M-B CDI-K Short Form as Screening Test in Children with Language Developmental Delay. Ann. Rehabil. Med..

[57] Can D.D., Ginsburg-Block M., Golinkoff R.M., Hirsh-Pasek K. (2013). A Long-Term Predictive Validity Study: Can the CDI Short Form Be Used to Predict Language and Early Literacy Skills Four Years Later?. J. Child. Lang..

[58] Jackson-Maldonado D., Marchman V.A., Fernald L.C.H. (2013). Short-Form Versions of the Spanish MacArthur-Bates Communicative Development Inventories. Appl. Psycholinguist..

[59] Vach W., Bleses D., Jørgensen R. (2010). Construction of a Danish CDI Short Form for Language Screening at the Age of 36 Months: Methodological Considerations and Results. Clin. Linguist. Phon..

